# A novel hereditary encephalopathy in four related Labrador Retrievers associated with a missense variant in the *ALDH5A1* gene

**DOI:** 10.1093/jvimsj/aalaf021

**Published:** 2026-01-21

**Authors:** Ellen Schofield, Thomas Butler, Rita Gonçalves, Louise Pettitt, Sophie Wyatt, Christopher A Jenkins, Joao Miguel De Frias, Bryan McLaughlin, Ed Pilkington, Rocio Orlandi, Cathryn S Mellersh, Paul Freeman, Helen Prunty, Sally L Ricketts, Georgina Harris

**Affiliations:** Canine Genetics Centre, Department of Veterinary Medicine, University of Cambridge, Cambridge CB3 0ES, United Kingdom; Queen’s Veterinary School Hospital, University of Cambridge, Cambridge CB3 0ES, United Kingdom; Department of Veterinary Science, Small Animal Teaching Hospital, University of Liverpool, Leahurst, Neston CH64 7TE, United Kingdom; Canine Genetics Centre, Department of Veterinary Medicine, University of Cambridge, Cambridge CB3 0ES, United Kingdom; Queen Mother Hospital for Animals, Royal Veterinary College, Hatfield AL9 7TA, United Kingdom; Canine Genetics Centre, Department of Veterinary Medicine, University of Cambridge, Cambridge CB3 0ES, United Kingdom; Queen Mother Hospital for Animals, Royal Veterinary College, Hatfield AL9 7TA, United Kingdom; Canine Genetics Centre, Department of Veterinary Medicine, University of Cambridge, Cambridge CB3 0ES, United Kingdom; Department of Veterinary Science, Small Animal Teaching Hospital, University of Liverpool, Leahurst, Neston CH64 7TE, United Kingdom; Department of Veterinary Science, Small Animal Teaching Hospital, University of Liverpool, Leahurst, Neston CH64 7TE, United Kingdom; Canine Genetics Centre, Department of Veterinary Medicine, University of Cambridge, Cambridge CB3 0ES, United Kingdom; Queen’s Veterinary School Hospital, University of Cambridge, Cambridge CB3 0ES, United Kingdom; Department of Chemical Pathology, Great Ormond Street Hospital NHS Trust, London WC1N 3BH, United Kingdom; Canine Genetics Centre, Department of Veterinary Medicine, University of Cambridge, Cambridge CB3 0ES, United Kingdom; Queen’s Veterinary School Hospital, University of Cambridge, Cambridge CB3 0ES, United Kingdom

**Keywords:** familial, seizures, panic-attack-like episodes, neurodegenerative, gene, dog

## Abstract

**Background:**

Hereditary neurodegenerative diseases occur in dogs, and a molecular diagnosis can be of value for treatment and prevention.

**Hypothesis/Objectives:**

To describe the clinical presentation of a novel encephalopathy in 4 related Labrador Retrievers. To identify a candidate causal variant for the disease using whole genome sequencing (WGS).

**Animals:**

Four related Labrador Retrievers presenting between 4 and 9 months of age.

**Methods:**

Case information and clinical workup were recorded for the 4 dogs in this case series. Two cases underwent WGS to identify candidate causal variants that were validated by genotyping Labradors related and unrelated to the cases, and by screening WGS of other breeds/canid species.

**Results:**

Clinical signs in cases included paroxysmal anxiety episodes, and focal and generalized epileptic seizures. Interictal clinical and neurological examinations were normal in all cases. Magnetic resonance imaging of the brain documented bilaterally symmetrical, T2-weighted image hyperintense, T1-weighted image isointense, and non-contrast-enhancing lesions within the lentiform nuclei, caudal colliculi, substantia nigra, and cerebellar nuclei. Investigations to exclude underlying nutritional, toxic, and metabolic causes were within normal limits. All cases had 2 copies of a missense variant in the aldehyde dehydrogenase 5 family member A1 (*ALDH5A1*) gene that segregated as expected in the family group and was absent in 70 unrelated Labradors and 2339 WGS of multiple breeds/canids.

**Conclusions and clinical importance:**

The prognosis of this novel hereditary encephalopathy in a family of Labradors appears fair with reasonable clinical response to administration of anti-seizure drugs. A missense variant in *ALDH5A1* has been identified as a candidate causal variant for the disease in these dogs.

## Introduction

Bilateral symmetrical polio-encephalopathy (BSPE) causing neurological disease in dogs is a rare presentation and is seen most frequently with degenerative, metabolic, and toxic diseases of the brain.^1^ However, the clinical presentation of BSPE could have some overlap with its more common counterpart, idiopathic epilepsy (IE). Hereditary neurodegenerative diseases are increasingly recognized in dogs, with the growing use of magnetic resonance imaging (MRI) contributing to an improved ability to identify focal lesions within the central nervous system. Hereditary BSPE is identified in several breeds, notably the Alaskan Husky^2–4^ and Yorkshire Terrier.^5^ In both breeds, this disease is caused by distinct, autosomal recessive, breed-specific mutations in exon 2 of the solute carrier family 19 member 3 (*SLC19A3*) gene that codes for the thiamine transporter 2.^3^^,^^6^ To the authors’ knowledge, BSPE has not been described in the Labrador Retriever.

A missense mutation in the aldehyde dehydrogenase 5 family member A1 (*ALDH5A1*) gene is described in a group of related Saluki dogs, resulting in Canine Succinic Semialdehyde Dehydrogenase Deficiency (SSADHD).^7^ Clinical signs included seizures, vocalization, and abnormal behavior. Bilateral symmetrical changes throughout the gray, and lesser so white, matter are found on MRI and histopathology.

Conversely, epilepsy in dogs is a common condition with an estimated prevalence of up to 0.82%.^8^ Epilepsy is often divided into idiopathic or symptomatic (secondary to a metabolic or underlying structural disease) etiologies.^9^ Labradors have a genetic predisposition to IE that is well documented^10^ with an estimated prevalence of 3.1% in a Danish population.^11^ Seizure semiology in Labradors with epilepsy is described as a mix of generalized and focal seizures, with most seizures reported to be generalized.^12^^,^^13^ Fear- or anxiety-related behavior as a manifestation of seizures is scarcely described in the veterinary literature, but most notably occurs in the Bull Terrier^14^ and Boerboel dogs.^15^ A small percentage of Labradors have “complex partial seizures” with psychomotor signs;^13^ however, panic or fear-related behavior as a manifestation of a focal seizure or seizure semiology has not yet been described in Labradors.

Our objectives were to describe the phenotype of a novel presumed hereditary disease, causing epilepsy with an unusual seizure semiology in a group of 4 related Labrador Retrievers, to allow better recognition of similar cases within the population and to conduct whole genome sequencing (WGS) of cases to identify the candidate causative variant for the disease. Our hypothesis was that this is a novel single-gene (likely autosomal recessive) disease with the underlying genetic defect segregating within the Labrador Retriever population.

## Materials and methods

### Clinical characterization of cases

**Figure 1:**
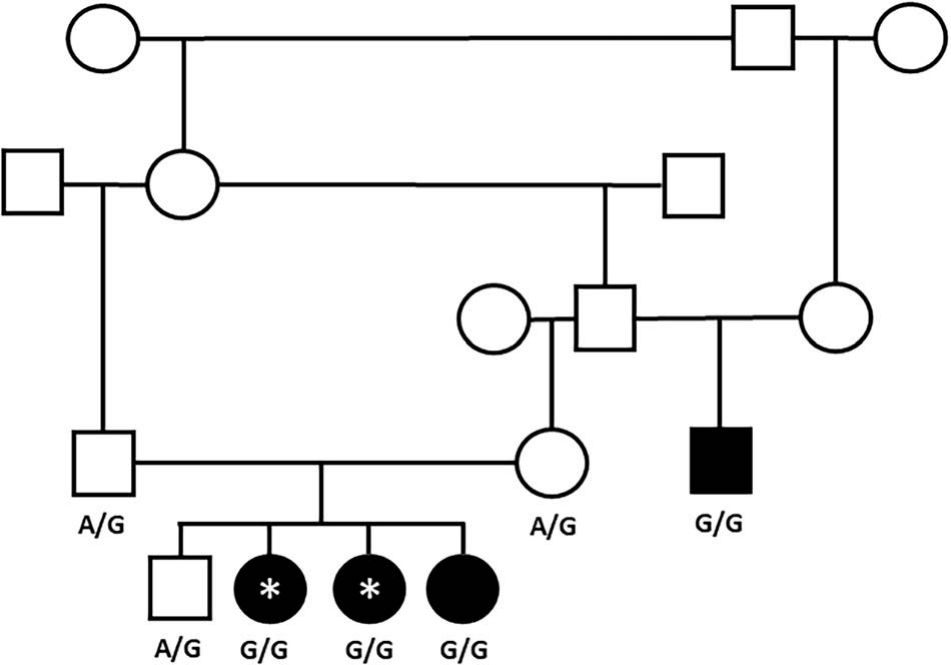
Pedigree showing the close genetic relationship of the affected dogs. Square = male, circle = female, and filled shape = affected dogs. WGS cases are shown with an asterisk. *ALDH5A1* variant genotypes are given below the individuals where available. Three additional littermates (1 female and 2 males) in the proband litter were not available for analysis and so are not shown on this pedigree. Abbreviations: WGS = whole genome sequencing; *ALDH5A1* = aldehyde dehydrogenase 5 family member A1.

In 2021, 3 female, purebred, UK Kennel Club-registered Labrador Retrievers from the same litter were presented to 3 separate UK tertiary referral hospitals for investigations into paroxysmal anxiety episodes (described as “panic attack-like” by the owners) and focal and generalized seizures: Case 1 to Queen’s Veterinary School Hospital, University of Cambridge; Case 2 to Small Animal Teaching Hospital, University of Liverpool (SATH); and Case 3 to the Queen Mother Hospital for Animals, Royal Veterinary College, London. Clinical and historical information was obtained from medical records of referring veterinarians and from client consultations. CBCs, routine serum biochemistry, serum cobalamin, brain MRI, and cerebrospinal fluid (CSF) analysis were performed in all dogs. Additional testing was performed at the individual clinician’s discretion. The sire of a fourth, related dog (Case 4) which had been presented to the SATH in 2018 with similar clinical signs, was also identified as the maternal grandsire of the litter (Figure 1).

### DNA analysis

Buccal swab samples were collected from Cases 1-3, their dam and sire, an unaffected littermate, and the related Case 4 with owner consent. Genomic DNA samples were extracted from buccal swabs using a QIAamp DNA Blood Midi Kit (Qiagen). Sanger sequencing was initially carried out to genotype Case 1, the unaffected dam of Cases 1-3, and an unrelated female Labrador Retriever control (with no report of neurological disease at DNA sample submission [aged > 9 years]), for the 2 *SLC19A3* variants previously associated with canine hereditary encephalopathy.^3^^,^^6^ PCR primers (forward: TCTTTCTTCCAGTGCCTTCCA, reverse: GCAAGCCACCTACACCTTAGT) were designed covering a 1065 base-pair region (Canine genome build UU_Cfam_GSD_1.0 chr25:40,614,842-40,615,906) that includes the exon in which the Alaskan Husky 4-base-pair insertion and Yorkshire Terrier 6-base-pair deletion/35 base-pair insertion are located. After PCR, sequencing was performed by Source Bioscience, using the PCR primers described above and 2 internal primers (forward: TGGAAGGTTCATCTGACACTGT, reverse: TATCCTTGGCCTCTGTCTGTG).

We conducted WGS using an Illumina 150 bp paired-end PCR-free DNA library preparation, with sequencing carried out on an Illumina NovaSeq instrument at the CRUK-CI Genomics Core, University of Cambridge, at a read depth of approximately 30×. Paired-end sequence data were aligned to the CanFam 4 canine genome assembly (German Shepherd Dog) using the Burrow-Wheeler Aligner Maximal Exact Matches algorithm,^16^ and variant calls were made using GATK v3.6.^17^

Follow-up of variants was conducted using Sanger sequencing of the 2 affected WGS dogs, an additional affected and unaffected littermate, the further affected relative, and the 2 unaffected parents (Figure 1). PCR primer sequences spanning the *ALDH5A1* variant were forward: CTCTATAGCTCGCAAACCAGG, reverse: ACTCTACTGCGAATCTCCCAG; and for the *CTU1* variant were forward: ATTGTGGTGTGGATGCATCTG, reverse: TCTTTCTCACTGTGACCACCG. Sequencing was again conducted by Source Bioscience. Additional genotyping of the *ALDH5A1* variant was done using a custom TaqMan SNP genotyping assay (forward: GGGAAGCACTTTGTACTGATCCT, reverse: CCAGTAGCTGTTGAGCCAGTAAAG, probes: TAGTGTCCAAAATTTC (wild type) and TAGTGTCCAGAATTTC (variant).

## Results

### Clinical phenotype

#### Case 1

An 8-month-old, female entire Labrador Retriever presented for investigation of increasing frequency and severity of paroxysmal (“panic attack-like”) anxiety episodes. These episodes were characterized by sudden-onset agitation, compulsive vocalization (barking, whining, and yelping), abnormal behaviors (frantic running and involuntary urination), and being poorly responsive to external stimuli. A several-hour post-ictal phase, during which time the dog was markedly unsettled, was reported (Video 1). Episodes started at 5 months of age with a frequency of once per month, increasing to every 48 h in the month prior to presentation.

**Figure 2:**
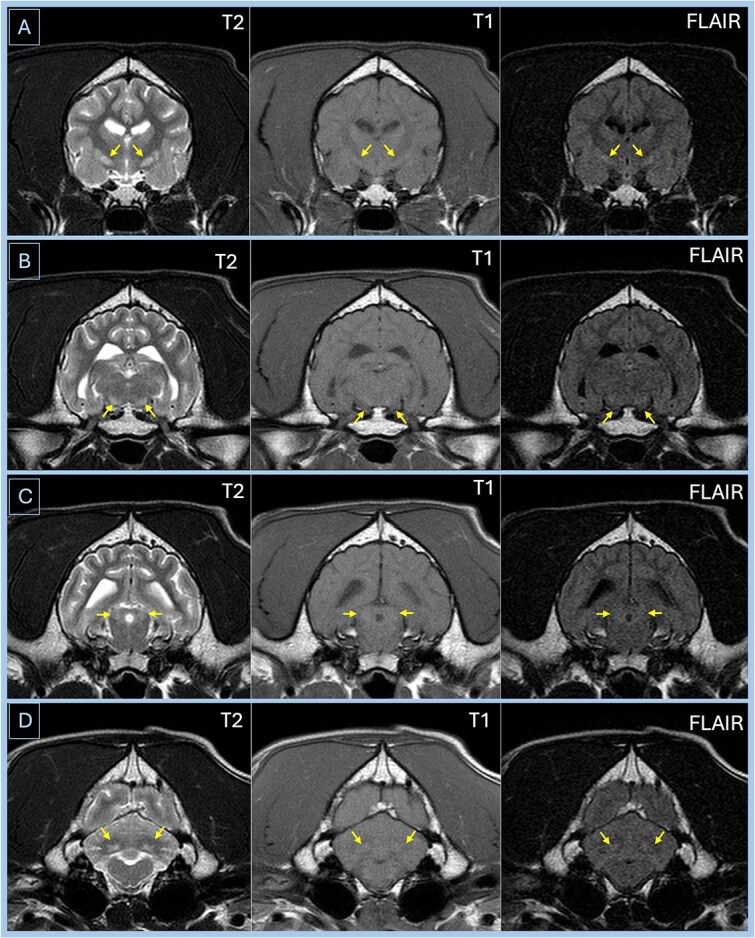
Case 1—Transverse MRI images of the brain of Case 1. Lesions are shown by the arrows. Image A is at the level of the lentiform nuclei and shows bilateral symmetrical lesions in the lentiform nuclei that are hyperintense on the T2-weighted and FLAIR images and isointense on the T1-weighted image. Image B is at the level of the substantia nigra and shows bilateral symmetrical lesions in substantia nigra that are hyperintense on the T2-weighted and FLAIR images and isointense to gray matter on the T1-weighted image. Image C is at the level of the caudal colliculi and shows bilateral symmetrical lesions in caudal colliculi that are hyperintense on the T2-weighted and FLAIR images and isointense to gray matter on the T1-weighted image. Image D is at the level of the cerebellar nuclei and shows bilateral symmetrical lesions in cerebellar nuclei that are hyperintense on the T2-weighted and FLAIR images and isointense to gray matter on the T1-weighted image. Abbreviation: FLAIR = fluid attenuated inversion recovery.

On presentation, no abnormalities were detected on physical and neurological examination. Serum biochemistry documented mild hyperphosphatemia (45 mg/dL [2.5 mmol/L], reference range [RR] 14.4-30.6 mg/dL [0.8-1.7 mmol/L]). Sediment examination of a free-catch urine sample documented pyuria and rod-shaped bacteriuria. Urinary metabolite testing documented the presence of hippurate/adipic acid, and the mucopolysaccharide spot test was slightly positive. CBC, cisternal CSF analysis, bile acid stimulation test, resting serum ammonia, serum trypsin-like immunoreactivity, folate and cobalamin concentrations, *Toxoplasma/Neospora* PCR on CSF, and CSF lactate concentration were normal. MRI of the brain documented focal, bilaterally symmetrical, well-defined, non-contrast-enhancing, T2-weighted image (T2w)/fluid attenuated inversion recovery (FLAIR) hyperintense, and T1-weighted image (T1w) isointense (relative to normal gray matter) regions within the lentiform nuclei, substantia nigra, caudal coliculi, and cerebellar nuclei (Figure 2).

The dog was treated with imepitoin (Pexion, Boehringer Ingelheim; 14 mg/kg twice daily PO) for management of the paroxysmal anxiety episodes and suspected seizure activity and amoxicillin-clavulanate (Clavaseptin, Vetoquinol; 15 mg/kg twice daily PO for 7 days) for the presumed urinary tract infection, which subsequently resolved (negative bacterial culture on a cystocentesis sample). After the development of 4 generalized tonic–clonic seizures within a 48-h period, the dog was administered levetiracetam (Keppra, GlaxoSmithKline; 27.5 mg/kg every 8 h PO). One month later, the frequency of the paroxysmal anxiety episodes and generalized seizure activity increased with focal seizures also noted, prompting initiation of administration of phenobarbital (Epiphen, Vetoquinol; 2.6 mg/kg twice daily PO). Focal seizures were described as lasting 1-2 min with ptyalism, defecation, and jaw-chomping. Repeat MRI was performed 3 and 12 months after the initial imaging, which documented no change, despite clinical progression of disease.

Further dose escalation in phenobarbital was required to control clinical signs (3.2 mg/kg twice daily PO), with serum phenobarbital concentrations confirmed to be within the therapeutic range (13.9 mg/L [60 μmol/L], RR 9.3-37.1 mg/L [40-160 μmol/L]). At the time of writing (32 months after initial presentation), generalized seizure frequency was drastically reduced, with a frequency of weekly paroxysmal anxiety episode occurrences, which were reported to last less than 30 s.

#### Case 2

An 8-month-old, female neutered Labrador Retriever presented for investigation of a 6-month history of increasing frequency of paroxysmal anxiety episodes, described as abnormal vocalization, frantic running, and hiding lasting 20-360 s. Recovery was described by the owner as taking approximately 30 min. Involuntary urination/defecation occasionally occurred during episodes. Episodes had increased in frequency from once in eight weeks to daily. No abnormalities were detected on physical and neurological examination. Serum biochemistry documented hyperphosphatemia (35.28 mg/dL [1.96 mmol/L], RR 14.4-28.8 mg/dL [0.8-1.6 mmol/L]) and the dog had a low serum total copper concentration (49.0 μg/dL [7.7 μmol/L], RR 57.3-95.5 μg/dL [9.0-15.0 μmol/L]) and was heterozygous for the *ATP7A* gene mutation, meaning the dog was at decreased risk of copper toxicosis or deficiency (Wilson’s and Menkes disease, respectively).^18^ CBC, urinalysis, cisternal CSF analysis, and serum cobalamin, thiamine, and caeruloplasmin were within normal limits. MRI of the brain documented bilaterally symmetrical, well-demarcated, T2w/FLAIR hyperintense, T1w hypointense (relative to normal gray matter), non-contrast-enhancing lesions within the lentiform nuclei, subthalamus, crus cerebri, and cerebellar nuclei (Figure S1). There were also bilaterally symmetrical, faintly T2w hyperintense lesions within the caudal colliculi.

The dog was treated with phenobarbital (2 mg/kg twice daily PO), with an initial good reduction in paroxysmal anxiety episode frequency. Progression in episode frequency and the development of generalized tonic–clonic epileptic seizures led to increased phenobarbital dosing (2.6 mg/kg twice daily PO) and the addition of levetiracetam (15 mg/kg twice daily PO). This led to an improvement in both seizure and episode control. At the time of writing (32 months after initial presentation), the dog was reported to have good seizure control (approximately every 2 months), with stable frequency of paroxysmal anxiety episodes (approximately once per week, often triggered by excitement) while receiving phenobarbital (3.5 mg/kg twice daily PO) and levetiracetam (22 mg/kg twice daily PO).

#### Case 3

A 9-month-old, female entire Labrador Retriever presented for investigation of a 2-month progressive history of paroxysmal anxiety episodes, manifesting as compulsive vocalization, frantic and compulsive running, and appearing fearful (Videos 2 and 3). Episodes were described as lasting minutes to hours, with increased generalized anxiety noted since the episodes started. Physical and neurological examinations were within normal limits. Serum biochemistry documented hyperphosphatemia (40.1 mg/dL [2.23 mmol/L], RR 14.4-28.8 mg/dL [.8-1.6 mmol/L]), hypoalbuminemia (24.3 g/L, RR 26.3-38.2 g/L) and hypoglobulinemia (19.3 g/L, RR 23.4-42.2 g/L). There was a mild serum hypocobalaminemia (174.0 ng/L, RR > 200 ng/L). CBC, urinalysis, cisternal CSF analysis, bile acid stimulation testing, abdominal ultrasonography, and serum thiamine concentration were within normal limits. MRI of the brain documented focal, well-demarcated, bilaterally symmetrical T2w/FLAIR hyperintense and T1w isointense (relative to normal gray matter), non-contrast-enhancing lesions in the lentiform nuclei, substantia nigra, and cerebellar nuclei, without associated mass effect (Figure S2).

The dog was treated with cobalamin supplementation (Cobaloplex, Protexin; 0.5 mg once daily PO) but developed increasing frequency and severity of paroxysmal anxiety episodes. One month following initial investigations, the dog suffered a generalized tonic–clonic epileptic seizure. Treatment was initiated with phenobarbital (1.9 mg/kg twice daily PO). After measurement of serum phenobarbital concentration (11.5 mg/L [49.4 μmol/L], RR 9.3-37.1 mg/L [40-160 μmol/L]), the dose was increased to 2.5 mg/kg twice daily. Subsequent increases in phenobarbital dosage were required to control paroxysmal anxiety episode activity. The dog continued to experience abnormal behavioral episodes 2-5 times per week of less than 5 min duration, which the owners managed through modification of the home environment (reducing stressful stimuli), 3 years after initial presentation.

#### Case 4

An 8-month-old, male entire Labrador Retriever presented for investigation of 3 generalized tonic–clonic epileptic seizures. The episodes were described as generalized, tonic–clonic seizures with hypersalivation and involuntary urination/defecation of approximately 2 min duration. Recovery lasted 2-3 h and involved pacing and circling to the right. Physical and neurological examinations were within normal limits. Serum biochemistry documented hyperphosphatemia (39.1 mg/dL [2.17 mmol/L], RR 14.4-28.8 mg/dL [.80-1.60 mmol/L]), hypoglobulinemia (22 g/L, RR 23.4-42.2 g/L) and total hypocalcemia (41.6 mg/dL [2.31 mmol/L], RR 42.5-51.1 mg/dL [2.36-2.84]). Ionized calcium was within normal limits. CBC, fasting serum ammonia, serum folate and cobalamin, cisternal CSF analysis, and metabolic panel (plasma amino acid and urinary organic acid testing) were within normal limits. MRI of the brain documented focal, well-demarcated, bilaterally symmetrical T2w/FLAIR hyperintense (relative to gray matter) lesions within the lentiform nuclei, substantia nigra, caudal colliculi, red nucleus, crus cerebri, and cerebellar nuclei (Figure S3).

The dog was administered phenobarbital (2 mg/kg twice daily PO) with dose escalation up to 3 mg/kg over the following 6 months. One additional generalized epileptic seizure was noted during this time. Repeat MRI was performed at re-examination 6 months later, documenting stable lesions as previously described, with some reduction in size of the lesions bilaterally within the cerebellar nuclei. Serum total copper concentration was low (47.8 μg/dL [7.5 μmol/L], RR 57.3-95.5 μg/dL [9.0-15.0]). Serum thiamine, blood gas analysis, serum caeruloplasmin and repeat CBC, resting ammonia and CSF analysis were within normal limits. The dog was clear of the Alaskan Husky encephalopathy *SLC19A3* mutation.^3^ The dog was re-examined 29 months later, with infrequent seizure activity (2 in 9 months) and on phenobarbital (3 mg/kg in the morning and 4 mg/kg in the evening PO). Repeat MRI documented stable T2w/FLAIR hyperintense, T1w hypointense (relative to gray matter) symmetrical lesions within the lentiform nuclei, substantia nigra, caudal colliculi, cerebellar peduncles, and cerebellar nuclei (Figure S4). At the time of writing, the dog was clinically well controlled on 4 mg/kg phenobarbital PO twice daily, with approximately 2 epileptic seizures per year, 5 years after initial presentation. Paroxysmal anxiety episodes were not reported in this dog, despite similar MRI findings to the related litter.

### Genetic analyses

#### Genotyping of known mutations

Case 1, the unaffected dam of Cases 1-3, and an unrelated control Labrador Retriever dog not reported to have neurological disease, were clear of the *SLC19A3* mutations described for Alaskan Husky and Yorkshire Terrier encephalopathy.^3^^,^^6^ Case 1 was also clear of a mutation recently identified for degenerative encephalopathy in Nova Scotia Duck Tolling Retrievers.^19^ These tests were performed as the MRI findings were considered suggestive for a metabolic, neurodegenerative disease, with bilaterally symmetrical abnormalities in regions of the brain associated with breed-specific conditions in the Alaskan Husky, Yorkshire Terrier, and Nova Scotia Duck Tolling Retriever.

#### WGS analysis

We compared WGS of the 2 Labrador cases to WGS of 352 dogs of 119 breeds (including nine Labradors) and 6 crossbreeds and identified 2 variants that were homozygous in both cases and absent from the control WGS. These were missense variants in the *CTU1* (CanFam 4 chr1:106101902; C > G; Glycine>Arginine) and *ALDH5A1* (CanFam 4 chr35:23980049; A > G; Lysine>Arginine) genes. Both variants were predicted to be deleterious using PolyPhen-2^20^ and Mutation Taster,^21^ and the *ALDH5A1* variant also using SIFT.^22^ The 2 variants were not present in the Dog10K WGS dataset that comprises 1987 purebred dogs, mixed-breed dogs, and other canids.^23^ The 2 Labrador cases were also clear of the *ALDH5A1* variant identified in the Saluki.^7^

#### Variant follow-up

We conducted initial validation of the 2 candidate variants by Sanger sequencing the 2 WGS cases, an additional affected littermate, an affected relative, an unaffected littermate, and the 2 unaffected parents. We did not develop a robust assay for the *CTU1* variant; Sanger sequences demonstrated that the variant did not segregate in the family group as expected (1 affected dog was homozygous for the C allele, an affected litter sibling homozygous for the G allele, 1 parent homozygous for the C allele, and the other for the G allele). Further *in silico* analysis of the region suggested that there might be additional copies of this DNA sequence in the canine genome downstream of the variant. We therefore excluded this variant from further study. The *ALDH5A1* variant segregated as expected for a disease with an autosomal recessive mode of inheritance (Figure 1). We performed further validation of the *ALDH5A1* variant by genotyping 70 Labradors collected for another study between 2009 and 2018 from the pet, show, and working population in the UK and later reported by their owners to be free of seizures in 2019/2020. The variant was not present in this set of dogs.

#### Targeted metabolic testing

Due to the identification of the candidate variant in the *ALDH5A1* gene and its documented link with canine SSADHD, Case 1 underwent targeted metabolic testing of the urine. The urine of Case 1 and a control dog (Parson Russell Terrier) were screened for urine organic acids that were seen to be increased in SSADHD.

Urinary organic acids were analyzed by gas chromatography mass spectrometry after oximation with hydroxylamine hydrochloride, liquid–liquid extraction with ethyl acetate and diethyl ether, and trimethylsilyl derivatization.

No metabolites associated with SSADH deficiency were detected in the control sample. In the affected sample, only 2,4-hydroxybutyric acid was detected, but this was not present in excess and is not specific for SSADH deficiency. Erythro and threo lactones of 4,5-dihydroxyhexanoic acid (DHHA) and 4-hydroxybutyrate were not detectable.

#### Comparative in silico protein analyses of the Labrador and Saluki variants

**Figure 3:**
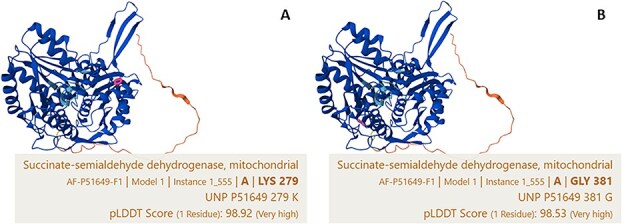
Structure of ALDH5A1 protein taken from the AlphaFold protein structure database (https://alphafold.ebi.ac.uk/entry/P51649) comparing the location of the Labrador K279R variant in the NAD-binding domain (A) and the Saluki G381D variant in the substrate-binding domain (B) of the protein (residues highlighted). Colors in the models and per-residue model confidence score (pLDDT score) denote the confidence level of structure (see https://alphafold.ebi.ac.uk/entry/P51649).

The Labrador variant is in exon 5 of the canine *ALDH5A1* gene as annotated in the CanFam 4 genome assembly, compared with the Saluki variant that lies within exon 7 (chr35:23984370). To assess where these variants are situated within the domains of the *ALDH5A1* protein, we utilized the AlphaFold Protein Structure Database^24^^,^^25^ (https://alphafold.ebi.ac.uk/entry/P51649). Figure 3 shows a comparison between the Labrador and Saluki variants that lie within different domains of the protein as defined by crystallography studies conducted by Kim et al.^26^

## Discussion

This case series and subsequent genetic analysis identifies a novel and hereditary encephalopathy in 4 related Labrador Retrievers. We detail the marked similarities in both clinical presentation and MRI findings across the 4 dogs and have found a missense mutation in the *ALDH5A1* gene that is shared in cases but has not been found in our sample sets of unaffected Labradors and WGS data of other purebred dog breeds, mixed-breed dogs, and species of canid. These findings and the absence of any metabolic or toxic causes, are highly suggestive of a genetic neurodegenerative disorder^27^ caused by the variant identified in the *ALDH5A1* gene.

A different missense mutation in *ALDH5A1* gene has been found to cause canine SSADHD in a group of related Saluki dogs.^7^ The *ALDH5A1* gene encodes for mitochondrial enzyme succinic semialdehyde dehydrogenase (SSADH), which is involved in catabolism of the inhibitory neurotransmitter gamma-aminobutyric acid.^26^^,^^28^ However, targeted metabolic testing of urine metabolites in our Labrador cases did not reveal any functional evidence of SSADHD; thus, as such, we cannot call the disease canine SSADHD. Nevertheless, similarities exist between the disease described in Salukis and our cases. These include cases presenting with generalized and focal seizures and paroxysmal vocalization.^7^ There were also significant similarities on MRI of the Salukis’ brains, documenting bilateral symmetrical changes mainly affecting the gray matter but also some white matter changes.^7^ Notable differences include an early onset of signs in the Saluki dogs (6-10 weeks), and sleep disturbances that were not reported in our cases. It is possible the variant found in our cases did not result in a non-functioning enzyme but rather a less effective form, giving rise to a less severe clinical phenotype. This theory is supported by the fact that the Saluki G381D variant is located adjacent to a published human disease variant (P382L)^29^ that has been predicted to cause a significant effect on protein stability, as amino acid 382 is located within a structurally important site.^26^ By contrast, the Labrador K279R variant is predicted to lie within the NAD+ binding domain of the protein and so might modulate its effect through reducing the efficiency of enzyme activity.^26^ However, this would require confirmation by cellular studies similar to those performed by Akaboshi et al. (2003) that confirmed a decrease in enzymatic activity of SSADH in the context of various known missense mutations in *ALDH5A1.*^29^

The prognosis of this disease in these Labradors appears fair, and while the clinical course is initially progressive, it can stabilize with appropriate anti-seizure medications (ASMs). The condition does not appear to be life-limiting, given that all 4 dogs described were alive at the time of writing with follow-up times from the onset of clinical signs ranging from 32 to 72 months. These findings are of particular importance given the breed predisposition for IE^10^^,^^13^^,^^30^ and the similarities in age of onset (6-9 months old), presentation (focal/generalized epileptic seizures with a normal inter-ictal neurological examination aside from paroxysmal anxiety episodes), and treatments (ASMs) between these 2 diseases.^30^^,^^31^ This novel condition might be more prevalent in the Labrador Retriever population than initially perceived, as the similarities with IE and the response to treatment with ASMs could lead to under-recognition of this disorder, particularly given that many dogs in general practice might be diagnosed with a Tier I confidence level for IE under the International Veterinary Epileptic Task Force consensus statement^32^ and therefore might not undergo MRI of the brain. Where financial implications of an MRI scan are a driving factor in preventing these investigations, a genetic test could be a useful cost-effective discriminator between these 2 conditions. It could also be useful to assess the disease allele frequency in a contemporary set of Labradors from the current UK population.

Characterization of novel clinical diseases and subsequent identification of their genetic cause are important to enable management and potential elimination of deleterious hereditary diseases in dogs. For example, WGS enabled the identification of a splice donor site mutation in the *sorting nexin 14* gene as the cause of autosomal recessive cerebellar cortical degeneration in the Hungarian Vizsla,^33^ leading to the development of a DNA test to try to eliminate the disease in the breed. Similar genetic sequencing studies have identified mutations associated with cerebellar ataxia in the Norwegian Buhund,^34^ BSPE in the Alaskan Husky^3^ and Yorkshire Terrier,^6^ spongy degeneration with cerebellar ataxia in Belgian Shepherd Dogs,^35^ and canine spongiform leukoencephalomyelopathy in Australian Cattle Dogs and Shetland Sheepdogs.^36^ Here we have identified a candidate causal variant showing strong segregation with the disease that will potentially lead to the elimination of this condition by the development of a genetic test.

In this case series, the authors have described the reported episodes as paroxysmal anxiety episodes, with the description of the episodes from owners as “panic-attack-like.” Similar seizure semiology has also been described in Boerboel^15^ and Bull Terrier^14^ with focal epilepsy with fear-related behavior. While we recognize that alternative etiologies to the episodes exist, for example, post-ictal behavioral change, hallucinatory seizures, and pain responses, we believe that the use of “panic-attack-like” or paroxysmal anxiety episode as descriptors is most appropriate, given owner descriptions and video footage of the episodes, in order to facilitate future identification of similar episodes in affected dogs by both members of the public and veterinarians.

There are several limitations to the current case series. The primary restricting factor is the lack of histopathological description of affected brain tissue to help elucidate the changes seen and compare them to the changes seen in SSADHD in Salukis^7^ and known polioencephalopathies, such as BSPE in the Alaskan Husky^2^ and Yorkshire Terrier.^5^ However, as all cases are currently still alive and clinically stable, this is not feasible but might be performed in the future via post-mortem examination. An additional limitation is that the dogs all had different investigations performed, due to their presentation to different hospitals, and the 2 cases attending the same center were seen 3 years apart. However, thorough investigations to rule out underlying nutritional, toxic, and metabolic causes were carried out in all cases with no significant findings. In addition, all dogs had a thorough metabolic workup and only hyperphosphatemia was found consistently across all cases. Due to the lack of associated abnormalities in calcium, the changes in phosphate were considered likely age-related and unrelated to the disease.

In conclusion, a novel encephalopathy has been identified in a group of related Labrador Retrievers, and WGS has identified a strong candidate variant in the *ALDH5A1* gene, making a hereditary condition most likely. The prognosis of the disease remains unknown but at present is considered fair, as the clinical course does not appear rapidly progressive and some response to ASMs has been observed.

## Supplementary Material

aalaf021_Supplemental_Files

## Off-label antimicrobial declaration

Authors declare no off-label use of antimicrobials.

## Institutional animal care and use committee or other approval declaration

Both clinical and genetic aspects of this study had ethical approval from the University of Cambridge Department of Veterinary Medicine Ethics and Welfare Committee (Nos. CR496, CR570, CR623, and CR869).

## Abbreviations

ALDH5A1: aldehyde dehydrogenase 5 family member A1
ASM: anti-seizure medication
ATP7A: ATPase copper transporting alpha
BSPE: bilateral symmetrical polio-encephalopathy
BWA-MEM: Burrow-Wheeler Aligner Maximal Exact Matches
CNS: central nervous system
CSF: cerebrospinal fluid
CTU1: cytosolic thiouridylase subunit 1
DNA: deoxyribonucleic acid
FLAIR: fluid attenuated inversion recovery
GATK: genomic analysis toolkit
IE: idiopathic epilepsy
MPS: mucopolysaccharide
MRI: magnetic resonance imaging
QMHA: Queen Mother Hospital for Animals
QVSH: Queen’s Veterinary School Hospital
RR: reference range
SLC19A3: solute carrier family 19 member 3
SNX14: sorting nexin 14
SSADHD: succinic semialdehyde dehydrogenase deficiency
T1w: T1 weighted image
T2w: T2 weighted image
TLI: trypsin-like immunoreactivity
WGS: whole genome sequence(d)/sequencing

## References

[1] Vandevelde M, Higgins R, Oevermann A. Degenerative diseases. In: Vandevelde M, Higgins R, Oevermann A, eds. Veterinary Neuropathology: Essentials of Theory and Practice. Wiley-Blackwell; 2012:157-189.

[2] Brenner O, Wakshlag JJ, Summers BA, de Lahunta A. Alaskan Husky encephalopathy – a canine neurodegenerative disorder resembling subacute necrotizing encephalomyelopathy (Leigh syndrome). Acta Neuropathol. 2000;100:50-62. 10.1007/s004010051192 10912920

[3] Vernau KM, Runstadler JA, Brown EA, et al. Genome-wide association analysis identifies a mutation in the thiamine transporter 2 (*SLC19A3*) gene associated with Alaskan husky encephalopathy. PLoS One. 2013;8:e57195. 10.1371/journal.pone.0057195 23469184 PMC3587633

[4] Vernau K, Napoli E, Wong S, et al. Thiamine deficiency-mediated brain mitochondrial pathology in Alaskan huskies with mutation in SLC19A3.1. Brain Pathol. 2015;25:441-453. 10.1111/bpa.12188 25117056 PMC4326624

[5] Baiker K, Hofmann S, Fischer A, et al. Leigh-like subacute necrotising encephalopathy in Yorkshire terriers: neuropathological characterisation, respiratory chain activities and mitochondrial DNA. Acta Neuropathol. 2009;118:697-709. 10.1007/s00401-009-0548-6 19466433

[6] Drögemüller M, Letko A, Matiasek K, et al. *SLC19A3* loss-of-function variant in Yorkshire terriers with Leigh-like subacute necrotizing encephalopathy. Genes (Basel). 2020;11:1215. 10.3390/genes11101215 33081289 PMC7650533

[7] Vernau KM, Struys E, Letko A, et al. A missense variant in ALDH5A1 associated with canine succinic semialdehyde dehydrogenase deficiency (SSADHD) in the saluki dog. Genes (Basel). 2020;11:1033. 10.3390/genes11091033 32887425 PMC7565783

[8] Erlen A, Potschka H, Volk HA, Sauter-Louis C, O'Neill DG. Seizure occurrence in dogs under primary veterinary care in the UK: prevalence and risk factors. J Vet Intern Med. 2018;32:1665-1676. 10.1111/jvim.15290 30216557 PMC6189390

[9] Berendt M, Farquhar RG, Mandigers PJJ, et al. International veterinary epilepsy task force consensus report on epilepsy definition, classification and terminology in companion animals. BMC Vet Res. 2015;11:182. 10.1186/s12917-015-0461-2 26316133 PMC4552272

[10] Jaggy A, Faissler D, Gaillard C, Srenk P, Graber H. Genetic aspects of idiopathic epilepsy in Labrador retrievers. J Small Anim Pract. 1998;39:275-280. 10.1111/j.1748-5827.1998.tb03650.x 9673903

[11] Berendt M, Gredal H, Pedersen LG, Alban L, Alving J. A cross-sectional study of epilepsy in Danish Labrador retrievers: prevalence and selected risk factors. J Vet Intern Med. 2002;16:262-268. 10.1892/0891-6640(2002)016<0262:acsoei>2.3.co;2 12041655

[12] Hülsmeyer V-I, Fischer A, Mandigers PJJ, et al. International veterinary epilepsy task Force’s current understanding of idiopathic epilepsy of genetic or suspected genetic origin in purebred dogs. BMC Vet Res. 2015;11:175. 10.1186/s12917-015-0463-0 26316206 PMC4552344

[13] Heynold Y, Faissler D, Steffen F, Jaggy A. Clinical, epidemiological and treatment results of idiopathic epilepsy in 54 labrador retrievers: a long-term study. J Small Anim Pract. 1997;38:7-14. 10.1111/j.1748-5827.1997.tb02977.x 9121134

[14] Dodman NH, Knowles KE, Shuster L, Moon-Fanelli AA, Tidwell AS, Keen CL. Behavioral changes associated with suspected complex partial seizures in bull terriers. J Am Vet Meed Assoc. 1996;208:688-691. 10.2460/javma.1996.208.05.688 8617623

[15] Stassen QEM, Grinwis GCM, van Rhijn NC, et al. Focal epilepsy with fear-related behavior as primary presentation in Boerboel dogs. J Vet Intern Med. 2019;33:694-700. 10.1111/jvim.15346 30580458 PMC6430876

[16] Li H, Durbin R. Fast and accurate short read alignment with burrows-wheeler transform. Bioinformatics. 2009;25:1754-1760. 10.1093/bioinformatics/btp324 19451168 PMC2705234

[17] Van der Auwera GA, Carneiro M, Hartl C, et al. From FastQ data to high-confidence variant calls: the genome analysis toolkit best practices pipeline. Curr Protoc Bioinformatics. 2013;43:11.10.1-11.10.33. 10.1002/0471250953.bi1110s43 PMC4243306 25431634

[18] Fieten H, Gill Y, Martin AJ, et al. The Menkes and Wilson disease genes counteract in copper toxicosis in Labrador retrievers: a new canine model for copper-metabolism disorders. Dis Model Mech. 2016;9:25-38. 10.1242/dmm.020263 26747866 PMC4728329

[19] Guo J, Bullock G, O'Brien DP, et al. An RB1CC1 missense variant in Nova Scotia duck tolling retrievers with degenerative encephalopathy. Genes (Basel). 2025;16:269. 10.3390/genes16030269 40149422 PMC11941761

[20] Adzhubei IA, Schmidt S, Peshkin L, et al. A method and server for predicting damaging missense mutations. Nat Methods. 2010;7:248-249. 10.1038/nmeth0410-248 20354512 PMC2855889

[21] Schwarz JM, Cooper DN, Schuelke M, Seelow D. MutationTaster2: mutation prediction for the deep-sequencing age. Nat Methods. 2014;11:361-362. 10.1038/nmeth.2890 24681721

[22] Ng PC, Henikoff S. Predicting deleterious amino acid substitutions. Genome Res. 2001;11:863-874. 10.1101/gr.176601 11337480 PMC311071

[23] Meadows JRS, Kidd JM, Wang GD, et al. Genome sequencing of 2000 canids by the Dog10K consortium advances the understanding of demography, genome function and architecture. Genome Biol. 2023;24:187. 10.1186/s13059-023-03023-7 37582787 PMC10426128

[24] Jumper J, Evans R, Pritzel A, et al. Highly accurate protein structure prediction with AlphaFold. Nature. 2021;596:583-589. 10.1038/s41586-021-03819-2 34265844 PMC8371605

[25] Varadi M, Anyango S, Deshpande M, et al. AlphaFold protein structure database: massively expanding the structural coverage of protein-sequence space with high-accuracy models. Nucleic Acids Res. 2022;50:D439-D444. 10.1093/nar/gkab1061 34791371 PMC8728224

[26] Kim Y-G, Lee S, Kwon O-S, et al. Redox-switch modulation of human SSADH by dynamic catalytic loop. EMBO J. 2009;28:959-968. 10.1038/emboj.2009.40 19300440 PMC2670868

[27] Patterson DF, Aguirre GA, Fyfe JC, et al. Is this a genetic disease? J Small Anim Pr. 1989;30:127-139. 10.1111/j.1748-5827.1989.tb01517.x

[28] Malaspina P, Picklo MJ, Jakobs C, Snead OC, Gibson KM. Comparative genomics of aldehyde dehydrogenase 5a1 (succinate semialdehyde dehydrogenase) and accumulation of gamma-hydroxybutyrate associated with its deficiency. Hum Genomics. 2009;3:106-120. 10.1186/1479-7364-3-2-106 19164088 PMC2657722

[29] Akaboshi S, Hogema BM, Novelletto A, et al. Mutational spectrum of the succinate semialdehyde dehydrogenase (*ALDH5A1*) gene and functional analysis of 27 novel disease-causing mutations in patients with SSADH deficiency. Hum Mutat. 2003;22:442-450. 10.1002/humu.10288 14635103

[30] Monteiro R, Adams V, Keys D, Platt SR. Canine idiopathic epilepsy: prevalence, risk factors and outcome associated with cluster seizures and status epilepticus. J Small Anim Pract. 2012;53:526-530. 10.1111/j.1748-5827.2012.01251.x 22835069

[31] Charalambous M, Brodbelt D, Volk HA. Treatment in canine epilepsy – a systematic review. BMC Vet Res. 2014;10:257. 10.1186/s12917-014-0257-9 25338624 PMC4209066

[32] Risio LD, Bhatti S, Muñana K, et al. International veterinary epilepsy task force consensus proposal: diagnostic approach to epilepsy in dogs. BMC Vet Res. 2015;11:148. 10.1186/s12917-015-0462-1 26316175 PMC4552251

[33] Fenn J, Boursnell M, Hitti RJ, et al. Genome sequencing reveals a splice donor site mutation in the *SNX14* gene associated with a novel cerebellar cortical degeneration in the Hungarian vizsla dog breed. BMC Genet. 2016;17:123. 10.1186/s12863-016-0433-y 27566131 PMC5002145

[34] Jenkins CA, Kalmar L, Matiasek K, et al. Characterisation of canine KCNIP4: a novel gene for cerebellar ataxia identified by whole-genome sequencing two affected Norwegian Buhund dogs. PLoS Genet. 2020;16:e1008527. 10.1371/journal.pgen.1008527 31999692 PMC7012447

[35] Mauri N, Kleiter M, Dietschi E, et al. A SINE insertion in *ATP1B2* in Belgian shepherd dogs affected by spongy degeneration with cerebellar ataxia (SDCA2). G3 (Bethesda). 2017;7:2729-2737. 10.1534/g3.117.043018 28620085 PMC5555477

[36] Li F-Y, Cuddon PA, Song J, et al. Canine spongiform leukoencephalomyelopathy is associated with a missense mutation in cytochrome b. Neurobiol Dis. 2006;21:35-42. 10.1016/j.nbd.2005.06.009 16026996

