# Supplementary material for: A novel hereditary encephalopathy in four related Labrador Retrievers associated with a missense variant in the ALDH5A1 gene
Source: J Vet Intern Med. 2026 Jan 21;40(1):aalaf021. doi: 10.1093/jvimsj/aalaf021 (PMC12881967; doi:10.1093/jvimsj/aalaf021)
Supplement: aalaf021_Supplemental_Files [file aalaf021_supplemental_files.zip › Supplemental_Information_aalaf021.docx]

**Supplemental Information**

**
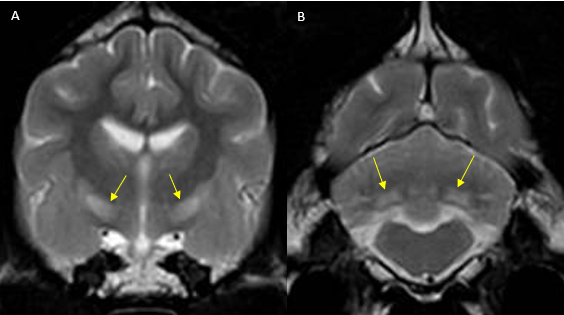
**

**Supplemental Figure 1** Case 2 – T2w images of the brain in transverse section. These reveal focal, bilaterally symmetrical hyperintensities (when compared to normal gray matter) affecting the lentiform nuclei (A) and cerebellar nuclei (B) (yellow arrows).


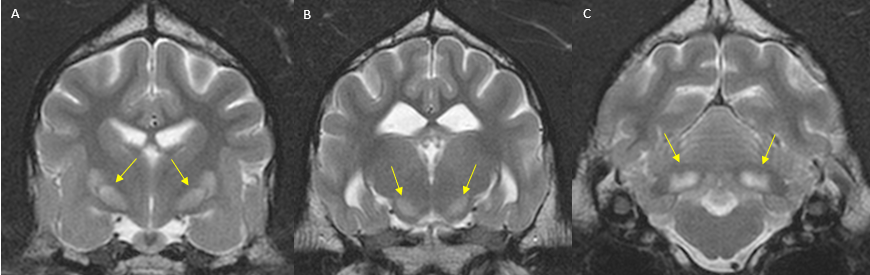


**Supplemental Figure 2** Case 3 – T2w images of the brain in transverse section. These reveal focal, bilaterally symmetrical hyperintensities (when compared to normal gray matter) affecting the lentiform nuclei (A), substantia nigra (B), and cerebellar nuclei (C) (yellow arrows).


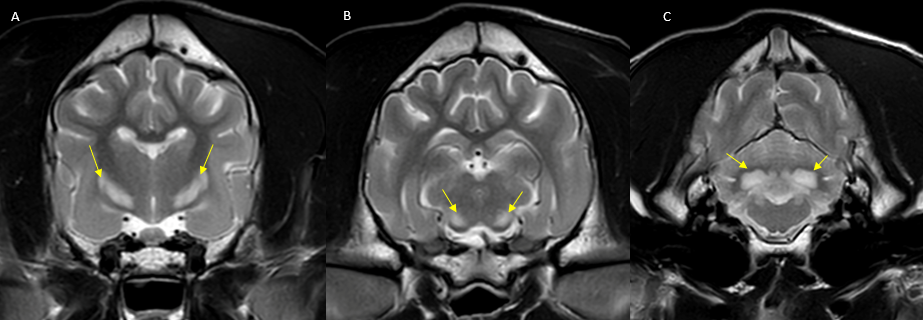


**Supplemental Figure 3** Case 4 (original MRI 2018) – T2w images of the brain in transverse section. These reveal focal, well demarcated, bilaterally symmetrical hyperintensities (when compared to normal gray matter) affecting the lentiform nuclei (A), substantia nigra (B), and cerebellar nuclei (C) (yellow arrows).


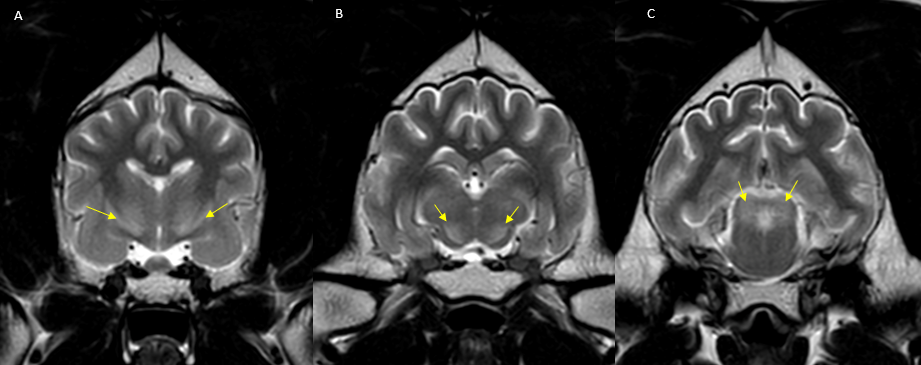


**Supplemental Figure 4** Case 4 (most recent MRI 2021) – T2w images of the brain in transverse section. These reveal focal, bilaterally symmetrical hyperintensities (when compared to normal gray matter) affecting the lentiform nuclei (A), substantia nigra (B), and caudal colliculi (C) (yellow arrows). This documented stable lesions as previously described.
